# Quality indicators of outpatient palliative care: A systematic review

**DOI:** 10.1016/j.apjon.2025.100718

**Published:** 2025-05-10

**Authors:** Sina Sharifi, Salman Barasteh, Akram Parandeh, Seyed Qasem Mousavi

**Affiliations:** a Student Research Committee, Baqiyatallah University of Medical Sciences, Tehran, Iran; b Nursing Care Research Center, Clinical Sciences Institute, Baqiyatallah University of Medical Sciences, Tehran, Iran; c Nursing Faculty, Baqiyatallah University of Medical Sciences, Tehran, Iran

**Keywords:** Quality indicators, Outpatient, Palliative care, End of life

## Abstract

**Objective:**

Outpatient palliative care is an integral part of providing services to patients with life-threatening conditions within health care systems. Identifying indicators of outpatient palliative care can help improve these services. Therefore, this study was conducted to identify quality indicators for the care of patients with life-limiting conditions in outpatient settings.

**Methods:**

This systematic review was conducted using Preferred Reporting Items for Systematic Reviews and Meta-Analyses (PRISMA) guidelines and included a search of databases such as PubMed, ProQuest, Science Direct, Web of Science, Scopus, and the Google Scholar search engine, with no time restrictions. Quality assessment was performed using the Appraisal of Indicators through Research and Evaluation (AIRE) tool. Final indicators were categorized based on a content analysis approach using conventional content analysis and the Donabedian model.

**Results:**

Six articles were selected for final analysis among the 659 identified articles. In this review, 32 quality indicators specific to outpatient palliative care were identified: 19 indicators were categorized under the process dimension, 11 under outcomes, and two under structure. These indicators were grouped into areas such as the patient-care provider relationship and mutual understanding (5 indicators), symptom and pain management (22 indicators), supportive care and referrals (5 indicators), and overall patient experience assessment (8 indicators).

**Conclusions:**

The findings of this study indicated that the identified indicators primarily focused on the physical and psychological aspects of outpatient palliative care. This review is the first to systematically categorize outpatient palliative care quality indicators and highlight priority areas for future development. There is a need to expand outpatient care quality indicators to include structural, cultural, and social dimensions to provide comprehensive, patient-centered palliative care.

## Introduction

Palliative care, as a specialized approach designed to improve the quality of life for patients with life-threatening illnesses, has gained significant attention worldwide for its comprehensive and patient-centered focus.^1^ Palliative care encompasses various domains, including physical, psychological, social, and spiritual care, all designed to address the unique needs of individuals and their families.^2^ With the aging global population and the rising prevalence of chronic diseases, the demand for palliative care services continues to grow.^3^ Recognizing the importance of providing holistic support to patients and families navigating complex health care challenges, palliative care has become an integral component of health care systems worldwide.^4^

Palliative care is delivered in various settings, each tailored to meet the diverse needs of patients with serious illnesses.^5^ One type of palliative care is hospital-based palliative care, typically provided in specialized units or through consultation teams, focusing on acute symptom management and coordination with other medical services.^6^ Another setting is home-based palliative care, where interdisciplinary teams offer medical, emotional, and practical support at the patient's residence, ensuring comfort and ease for the patient.^7^ Additionally, outpatient palliative care involves patients visiting clinics for regular appointments with palliative care specialists. This approach reduces hospital visits, improves continuity of care, and allows for timely interventions and symptom management.^8^

Outpatient palliative care has undergone significant development worldwide, with many countries recognizing its importance in improving the quality of life for patients with serious illnesses. Studies conducted in countries such as the United States, Belgium, the United Kingdom, and Australia have examined comprehensive outpatient palliative care programs.^5^ These settings provide a wide range of services, including symptom management, psychological support, advanced care planning, and coordination with other health care providers.^9^ The development of outpatient palliative care has been marked by the establishment of specialized clinics and its increasing integration into existing health care systems.^10^ Overall, the growth of outpatient palliative care reflects a rising awareness of its benefits, including reduced hospitalizations, improved symptom control, and increased satisfaction among patients and their families. By offering care in a less intensive environment, outpatient palliative care helps maintain patients’ independence, enables more personalized and continuous care, and ultimately leads to better outcomes and an enhanced quality of life.^11^

Focusing specifically on outpatient palliative care services requires attention to certain unique characteristics.^12^ Outpatient palliative care is designed for individuals who prefer to receive care in their homes or community settings. This type of care offers flexibility and convenience while promoting autonomy and independence.^13^ Unlike inpatient settings, outpatient palliative care relies on effective coordination among interdisciplinary team members, collaboration with primary care providers, and seamless transitions between different care settings.^14^ A clear understanding of the differences and challenges associated with outpatient palliative care is essential for optimizing service delivery and meeting the diverse needs of patients and families outside the traditional hospital environment.^15^

On the other hand, ensuring the delivery of high-quality palliative care is crucial for achieving optimal outcomes for patients and their families.^16^ Quality care in palliative care settings not only reduces physical symptoms but also addresses emotional distress, strengthens communication, promotes shared decision-making, and fosters dignity and respect.^17^ High-quality palliative care is characterized by access, continuity, coordination, and responsiveness to the evolving needs and preferences of patients and their families throughout the course of illness.^18^^,^^19^

Identifying and using quality indicators plays a key role in assessing and improving the delivery of palliative care services.^9^ Quality indicators serve as measurable markers that reflect the effectiveness, safety, timeliness, and patient-centeredness of care.^20^ By establishing and evaluating these indicators, health care providers can monitor performance, identify areas for improvement, and implement evidence-based practices to enhance overall care delivery.^21^

The expansion of palliative care across various health care settings and among different patient populations has highlighted the critical importance of evaluating and improving the quality of care provided.^15^ Despite this recognition, there remains a significant gap in our understanding of palliative care quality, particularly for patients receiving these services in the early stages of their illness in outpatient clinic settings.^22^ A systematic review conducted in 2023 identified 109 quality indicators specific to palliative care in intensive care units.^23^ However, to date, no comprehensive review has been conducted to examine quality indicators specific to outpatient clinics. Given that outpatient palliative care models may differ significantly from care provided in other settings,^24^ there is an urgent need to clarify the essential criteria for evaluating and improving care quality in this domain.

As a result, conducting a systematic review to identify quality indicators specific to outpatient palliative care is an important effort with far-reaching implications. By combining existing evidence and clarifying knowledge gaps, this study aims to identify quality indicators for outpatient palliative care and make them accessible to policymakers, health care providers, researchers, and stakeholders, regarding the essential criteria for evaluating and improving the quality of outpatient palliative care services.

## Methods

### Study design

To ensure methodological accuracy and minimize the risk of bias, this systematic review adhered to the guidelines set forth by the Preferred Reporting Items for Systematic Reviews and Meta-Analyses (PRISMA).^25^ The protocol of this systematic review was approved by the Institutional Ethics Committee of Baqiyatallah University of Medical Sciences (Approval No. IR.BMSU.BAQ.REC.1403.067).

### Research question

This systematic review was guided by the following research questions:

(1) What quality indicators are currently used to evaluate outpatient palliative care?
(2) How can these indicators be categorized into the domains of the Donabedian model (structure, process, and outcome)?
(3) What is the methodological quality of the studies that developed or reported these indicators?

The PICO framework includes:

(1) Population: All patients requiring outpatient palliative care. Intervention: Outpatient palliative care indicators. Comparison: Not applicable (as this review aims to identify and categorize indicators specific to outpatient palliative care, rather than compare them to other settings).
(2) Outcome: Identification and categorization of existing outpatient palliative care quality indicators, classification of these indicators based on the Donabedian model, and assessment of the methodological quality of the included studies.

### Search strategy

In May 2024, a search was conducted across five major databases: PubMed, ProQuest, ScienceDirect, Web of Science, and Scopus, along with the Google Scholar search engine. The search terms were selected in collaboration with a university librarian and included keywords such as “palliative care,” “quality indicators,” “quality measurement,” and “outpatient” (a full list of the keywords is provided in Supplementary Table S1). To broaden the search scope, no restrictions were applied regarding the year of publication. The search results were then imported into EndNote version 6, a reference management software, for effective organization and referencing. Additionally, we manually reviewed reference lists and other relevant sources to identify additional articles related to this study.

### Inclusion and exclusion criteria

The inclusion criteria for selecting articles were as follows: studies that examined quality indicators in outpatient palliative care. Quality indicators refer to evidence-based, measurable items that assess the structures, processes, and outcomes of health care.^19^ This study included research that examined both quality indicators and quality measures in outpatient palliative care. Studies of all patients in need of outpatient palliative care were considered, regardless of specific patient populations. Various study designs, including quantitative methods, mixed methods, RCTs, observational studies, and qualitative studies, were eligible for inclusion. Additionally, only studies published in English with full-text access focusing on outpatient palliative care were included. In contrast, review articles and studies written in languages other than English were excluded based on the exclusion criteria.

### Study selection

Duplicate studies identified across various databases were initially removed to ensure the inclusion of only unique and relevant articles. The screening process began with a review of titles and abstracts to assess their alignment with the inclusion criteria. Next, the full texts of the articles were reviewed, and irrelevant studies were excluded based on the predefined inclusion and exclusion criteria. If full-text access to eligible articles was unavailable, or if the data were unpublished, incorrect, or ambiguous, an email was sent to the corresponding author, followed by up to three additional emails at intervals of one–10 days. Articles were excluded if no response was received after three attempts. To minimize bias, two independent researchers conducted all steps of the analysis and data extraction. In cases of disagreement between the researchers, a third reviewer was consulted to ensure accuracy and consistency in the results.

### Quality assessment of studies

The Appraisal of Indicators through Research and Evaluation (AIRE) tool was used to assess the identified studies. AIRE is a validated tool for the critical evaluation of quality indicators and has been widely applied in various care settings. The AIRE tool comprises 20 items across four domains: 1) purpose, relevance, and organizational context; 2) stakeholder involvement; 3) scientific evidence; and 4) additional evidence, formulation, and usage.^26^ Two researchers independently assessed the quality indicators using the information provided in the studies. Each AIRE item was evaluated using a 4-point Likert scale, ranging from 1 (“strongly disagree or no information available”) to 4 (“strongly agree”). Discrepancies of more than one point between the researchers’ evaluations were resolved through discussion among the authors. Standardized scores for each domain were calculated, ranging from 0% to 100%, with higher scores indicating better quality. Since the aim of the study was to report all potential quality indicators for outpatient palliative care, no studies were excluded based on their quality.

### Data extraction

Two researchers independently conducted data extraction using a predefined checklist. The checklist included the primary author's name, year of publication, geographic location of the study, sample size, study type, and characteristics or quality indicators of outpatient palliative care.

### Data analysis

After completing data extraction, the indicators and measures were categorized using a two-step approach: first, a conventional content analysis and then the Donabedian model. In the conventional content analysis method, categories are derived directly from the data in an inductive manner, allowing for the natural emergence of themes and patterns without imposing preconceived frameworks.^27^ The Donabedian model comprises three domains: structure, process, and outcome.^28^ Structures of care include the availability of physical resources, personnel, and the provider's ability to meet patient needs. Processes of care involve the actions taken to address these needs. Outcomes reflect the observed effects of the provider's actions, as seen in changes in the patient's condition.

## Results

**Fig. 1:**
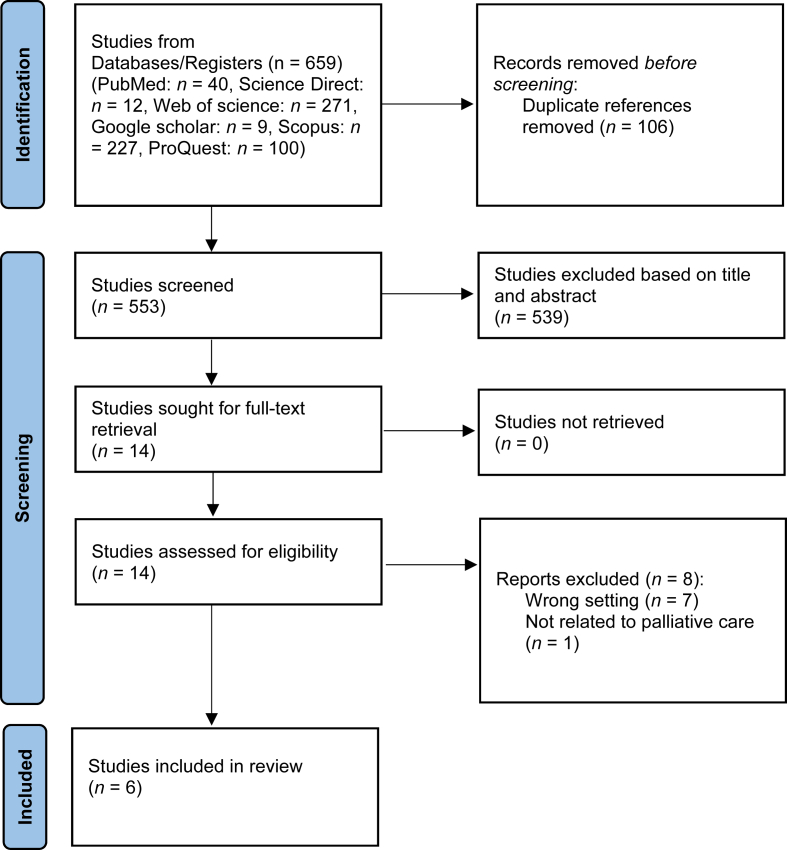
The flowchart on the stages of including the studies in the systematic review (PRISMA 2020). PRISMA, Preferred Reporting Items for Systematic Reviews and Meta-Analyses.

A total of 659 articles were initially identified and managed using EndNote software version 6, where 106 duplicate articles were removed. Titles and abstracts of the remaining articles were screened based on the inclusion and exclusion criteria, leading to the exclusion of 539 articles. During the full-text review stage, 14 articles were assessed, and eight were excluded based on the criteria. Ultimately, six articles met the eligibility criteria and were included in the final analysis. The PRISMA flowchart in Fig. 1 illustrates the search and selection process, and detailed information about the six included articles is provided in Table 1.

**Table 1:** Summary of characteristics of included studies in the systematic review.

Author/ year	Country	Method	Population (sample size)	Setting	Disease	Aim	Indicator
Ahluwalia et al., 2022^34^	USA	A mixed-method	2804 respondents in 44 ambulatory palliative care programs	Ambulatory palliative care	All patients	Develop and validate a patient-reported measure of the ambulatory palliative care experience called “feeling heard and understood"	1. Feeling heard and understood by the provider and team 2. Perceiving that the provider and team prioritize the individual's best interests when making care recommendations 3. Feeling seen as a person by the provider and team, not solely as someone with a medical problem 4. Feeling understood by the provider and team regarding what is important in the individual's life
Campion et al. 2011^29^	USA	Observational study	41 sites, with 92 physicians	Outpatient oncology	Cancer	Advancing performance measurement of palliative care in oncology	1. Pain assessed on either of the last two visits before death 2. Pain assessed appropriately 3. Pain intensity quantified on either of the last two visits before death 4. Plan of care for moderate/severe pain documented on either of the last two visits before death 5. Dyspnea assessed on either of the last two visits before death 6. Dyspnea addressed on either of the last two visits before death and appropriately 7. Hospice enrollment or palliative care referral 8. Chemotherapy administered within the last 2 weeks of life
Dy et al. 2010^30^	USA	Mixed methods	356 advanced cancer patients in two settings	Both outpatient and inpatient settings	Cancer	Evaluate feasibility, inter-rater reliability, and validity of quality indicators	1. Screening for pain intensity during cancer-related outpatient visits 2. Assessing effectiveness of pain treatment changes at subsequent outpatient visits 3. Regular assessment of nausea/vomiting for patients undergoing chemotherapy or with gastrointestinal/abdominal cancer 4. Evaluating effectiveness of antiemetic treatment for nausea/vomiting 5. Assessing fatigue during chemotherapy visits 6. Screening for anorexia/dysphagia during initial or follow-up visits for specific cancer types 7. Evaluating improvement in anorexia after appetite stimulant treatment 8. Offering symptomatic management for new/worsening dyspnea in lung cancer or advanced cancer patients
Lorenz et al. 2009^31^	USA	Mixed methods	9 expert panelists	Both outpatient and inpatient settings	Cancer	Develop a comprehensive set of quality indicators for supportive cancer care	1. Screening for the presence or absence and intensity of pain 2. Assessing effectiveness of pain treatment changes at subsequent outpatient visits 3. Assessment of nausea/vomiting for patients at every visit 4. Post-chemotherapy communication plan for patients undergoing moderately to highly emetic chemotherapy regimens 5. Assessing underlying causes for nausea or vomiting in outpatients not receiving chemotherapy or radiation 6. Evaluating the effectiveness of antiemetic treatment for nausea or vomiting in outpatients not undergoing chemotherapy or radiation 7. Assessment of the presence or absence of fatigue 8. Assessment for the presence or absence of anorexia or dysphagia 9. Offering symptomatic management or treatment for new or worsening dyspnea in outpatients 10. Referring outpatients with advanced cancer for hospital based palliative care within six months before expected death
Rollison et al., 2022^32^	USA	Qualitative/ Cognitive interview	25 outpatient palliative care patients and caregivers	Outpatient setting	Cancer, progressive MS, diabetes and heart disease, chronic regional syndrome, multiple kidney transplants, Crohn's disease	Develop survey items assessing patients' experiences of outpatient palliative care	1. Heard and understood (thinking about your overall experience with this provider and team in the last 6 months, how true is the following statement: I Felt heard and understood by this provider and team.) 2. Pain help (in the last 6 months, have you ever had pain?, In the last 6 months, did you desire assistance from this provider and team? In the last 6 months, did you get as much help as you wanted for your pain from this provider and team?)
Walling et al. 2023^33^	USA	Multi-method	A 30-member technical expert	Outpatient setting	–	Developing and testing patient-reported measures in outpatient setting	1. Feeling heard and understood (Q1: I Felt heard and understood by this provider and team, Q2: I Felt this provider and team put my best interests first when making recommendations about my care, Q3: I Felt this provider and team saw me as a person, not just someone with a medical problem, Q4: I Felt this provider and team understood what is important to me in my life.) 2. Receiving desired help for pain (in the last six months, have you ever had pain? In the last six months, did you want help from this provider and team for this pain? In the last six months, did you get as much help as you wanted for your pain from this provider and team?)

### Overview of indicators

All quality indicators were developed in the United States. Three studies specifically focused on cancer,^29^, ^30^, ^31^ while two studies included both cancer and other illnesses, such as diabetes, advanced multiple sclerosis, chronic kidney disease, and heart disease. The studies included a total patient population of 3185 and 39 experts.

The methods primarily consisted of literature reviews and expert consensus (4 mixed-method studies). One study utilized cognitive testing,^32^ while another employed data collection, pretesting, piloting, and validation phases.^33^ Four studies were conducted exclusively in outpatient settings, while two included both outpatient and inpatient settings.^30^^,^^31^ However, these two studies reported indicators separately for outpatient and inpatient environments; therefore, only the outpatient indicators were included in our analysis.

### Indicators based on content analysis

#### Patient–Provider Relationship and Mutual Understanding

This category includes five indicators that focus on the quality of interactions between patients and health care providers. These indicators assess whether patients feel heard, understood, and prioritized during their care. For example, indicators such as “feeling seen as a person, not just a medical case”^34^ and “feeling that life priorities are understood by the care team”^33^ highlight the importance of empathy and personalized care in outpatient palliative care settings. These measures reflect patients' subjective experiences and aim to capture the human aspects of health care delivery. Table 2 provides an overview of the indicators based on various classifications.

**Table 2:** Indicators classified by Palliative Care Domains.

Category	Indicators
Patient–provider relationship and understanding	Feeling heard and understood by provider and team∗ (outcome) Being heard and understood∗ (outcome) Perceived prioritization of individual benefits by provider and care team∗ (outcome) Feeling seen as a person by provider and team, not just as someone with a medical issue∗ (outcome) Feeling that life priorities are understood by provider and care team∗ (outcome)
Symptom and pain management	Screening for the presence or absence of pain severity (process) Assessment of the effectiveness of pain management adjustments in follow-up outpatient visits (outcome) Pain assessment during each of the last two visits before expected death (process) Proper pain assessment (process) Severity assessment of pain during each of the last two visits before expected death (process) Care plan for moderate/severe pain recorded during each of the last two visits before expected death (process) Screening for pain severity during cancer-related outpatient visits (process) Patient self-reporting of pain (in the past 6 months) (outcome)∗ Adequate pain relief assistance from the care team in the past 6 months (outcome)∗ Desired help for pain (in the past 6 months, have you experienced pain?) (outcome)∗ Request for assistance from the care team for pain in the past 6 months (outcome) Assessment of underlying causes of nausea or vomiting in outpatients not receiving chemotherapy or radiotherapy (process) Effectiveness assessment of anti-nausea/vomiting treatment for outpatients not on chemotherapy or radiotherapy (process) Regular assessment of nausea/vomiting in patients receiving chemotherapy or with gastrointestinal/abdominal cancer (process) Effectiveness assessment of anti-nausea treatment for nausea/vomiting (process) Assessment of presence or absence of anorexia or dysphagia (process) Screening for anorexia/dysphagia during initial or follow-up visits for specific cancer types (process) Assessment of improvement in anorexia following appetite stimulant treatment (process) Symptomatic management or treatment of new or worsening dyspnea in outpatients (process) Dyspnea assessed during each of the last two visits before expected death (outcome) Symptomatic management of new or worsening dyspnea in patients with lung cancer or advanced cancer (process) Assessment of presence or absence of fatigue (process)
Supportive care and referrals	Chemotherapy administered within the last 2 weeks of life (process) Hospital admission or palliative care referral (structural) Outpatient referral for advanced cancer patients to hospital palliative care within six months before expected death (structural) Post-chemotherapy communication plan for patients on high-risk nausea chemotherapy regimens (process) Post-chemotherapy communication plan for patients on high-risk diarrhea chemotherapy regimens (process)
Overall patient experience assessment	Patient self-reporting of pain (in the past 6 months) (outcome)∗ Adequate pain relief assistance from the care team in the past 6 months (outcome)∗ Desired help for pain (in the past 6 months, have you experienced pain?) (outcome)∗ Feeling heard and understood by provider and team (outcome) Being heard and understood (outcome) Perceived prioritization of individual benefits by provider and care team (outcome) Feeling seen as a person by provider and team, not just as someone with a medical issue (outcome) Feeling that life priorities are understood by provider and care team (outcome)

Indicators marked with an asterisk (∗) appear in two categories.

#### Symptom and pain management

This category encompasses the largest number of indicators—a total of 22—covering critical aspects of symptom evaluation, pain management, and relief strategies. These indicators range from the initial screening of pain severity and its accurate assessment to more specific criteria, such as “effectiveness of anti-nausea treatments”^31^ and “management of new or worsening shortness of breath”.^29^ The indicators include both process measures, such as “care plans for moderate or severe pain,” and outcome measures, such as “patient-reported pain relief.” These indicators emphasize timely and effective symptom control to ensure an improved quality of life for patients.

#### Supportive care and referrals

This category includes five indicators that assess the accessibility and timeliness of supportive services and referrals in outpatient palliative care. Examples include “hospital admission or referral to palliative care” and “outpatient referral for patients with advanced cancer within six months of expected death”.^31^ These indicators highlight the importance of coordinated care transitions and ensure that patients receive appropriate support as their condition progresses. Other criteria, such as “communication plans after chemotherapy for high-risk nausea or diarrhea regimens,” focus on reducing complications and maintaining continuity of care.

#### Overall patient experience assessment

This category includes eight indicators that evaluate the overall care experience from the patient's perspective. Indicators such as “adequate assistance in pain relief by the care team” and “receiving expected help for pain in the last six months” reflect the effectiveness of care in addressing patient needs. This category overlaps with aspects of the patient-provider relationship and emphasizes shared decision-making and satisfaction with care outcomes. These criteria aim to capture a comprehensive understanding of the patient's experience to ensure alignment with their expectations and values (Table 2).

### Indicators based on the donabedian model

Based on the Donabedian model, the indicators of quality in outpatient palliative care are classified into three categories: Structure, Process, and Outcome. The majority of the indicators fall under the Process category (number of processes ​= ​19). In the Process domain, pain assessment and management, management of gastrointestinal issues, and psychological aspects of care are of particular importance. Indicators such as Screening for the presence or absence of pain severity, Assessment of the effectiveness of pain management adjustments in follow-up outpatient visits, and Proper pain assessment focus on pain assessment and management, highlighting how care processes are involved in diagnosing, monitoring, and improving pain in patients. Additionally, indicators such as Assessment of presence or absence of anorexia or dysphagia and Symptomatic management or treatment of new or worsening dyspnea in outpatients address the management of gastrointestinal and respiratory issues. Psychological aspects are also reflected in indicators like Feeling heard and understood by provider and team, being seen as a person by provider and team, and Perceived prioritization of individual benefits by provider and care team, which emphasize the importance of the patient-provider relationship in the care process.

The Structure category includes indicators related to the infrastructure and organization of care, but there are only two such indicators in the studies reviewed, namely Hospital admission or palliative care referral and Outpatient referral for advanced cancer patients to hospital palliative care within six months before expected death. These indicators highlight the importance of access to palliative care services and the availability of appropriate infrastructure to ensure high-quality care in the final stages of life.

Finally, in the Outcome category, there are 11 indicators that address the results of care and its effects on patients (Table 2). Indicators such as Patient self-reporting of pain (in the past 6 months) and Adequate pain relief assistance from the care team in the past 6 months focus on the direct outcomes of pain management, while indicators like Feeling heard and understood by provider and team and Feeling that life priorities are understood by provider and care team reflect the psychological and emotional outcomes of care, addressing the impact of care on the patient's overall experience and quality of life.

### Quality assessment of included studies

The quality assessment of outpatient palliative care quality indicators, using the AIRE tool, revealed significant variations across different domains (Table 3). In the domain of “Purpose, relevance, and organizational context”, overall scores ranged from 80% to 92%, indicating that most studies provided clear and precise explanations of the objectives and organizational contexts of the indicators. Lorenz et al.^31^ and Dy et al.^30^ achieved the highest scores in this domain, with 90% and 92%, respectively. For “Stakeholder involvement”, overall scores ranged from 50% to 79%, reflecting a need for improvement in ensuring stakeholder participation during the indicator development process. Rollison et al.^32^ and Ahluwalia et al.^34^ received the lowest scores in this domain. In the domain of “Scientific evidence”, scores varied from 50% to 91%, highlighting differences in the use of systematic methods for gathering and incorporating scientific evidence. Dy et al.^30^ performed the best in this domain, with a score of 91%. Finally, for “Additional evidence, formulation, and use”, scores ranged from 59% to 83%, reflecting varying levels of detail and precision in defining the target population and incorporating risk adjustment strategies. Ahluwalia et al.^34^ scored 81%, while Campion et al.^29^ achieved the highest score of 83% in this domain.

**Table 3:** Critical appraisal of Methodological Quality of Quality Indicator.

AIRE domain	Walling et al., 2023	Rollison et al., 2022	Lorenz et al., 2009	Dy et al., 2010	Campion et al., 2011	Ahluwalia et al., 2022
**Purpose, relevance and organizational context**	**80%**	**87%**	**90%**	**92%**	**90%**	**87%**

1. The purpose of the indicator is described clearly	3.5	4	4	4	3.5	4
2. The criteria for selecting the topic of the indicator are described indetail	3	3	3	4	4	3
3. The organizational context of the indicator is described in detail	3	4	4	3	4	3.5
4. The quality domain the indicator addresses is described indetail	3	3.5	4	4	3	4
5. The health-care process covered by the indicator is described and defined in detail	3.5	3	3	3.5	3.5	3
**Stakeholder involvement**	**79%**	**58%**	**62%**	**79%**	**67%**	**50%**
6. The group developing the indicator includes individuals from relevant professional groups	3.5	2.5	3	3.5	3	2
7. Considering the purpose of the indicator, all relevant stakeholders have been involved at some stage of the development process	3.5	2	2.5	3	3	2
8. The indicator has been formally endorsed	2.5	2.5	2	3	2	2
**Scientific evidence**	**70%**	**50%**	**79%**	**91%**	**87%**	**54%**
9. Systematic methods were used to search for scientific evidence	2.5	1	3.5	4	4	1
10. The indicator is based on recommendations from an evidence-based guideline or studies published in peer-reviewed scientific journals	3	3	3	3	3	3
11. The supporting evidence has been critically appraised	3	2	3	4	3.5	2.5
**Additional evidence, formulation, usage**	**80%**	**59%**	**68%**	**77%**	**83%**	**81%**
12. The numerator and denominator are described in detail	2.5	2	2.5	3	3	2
13. The target patient population of the indicator is defined clearly	3	3	3	3	4	4
14. A strategy for risk adjustment has been considered and described	2.5	2	2.5	2	3	3
15. The indicator measures what it is intended to measure	3.5	3	3	4	3.5	3
16. The indicator measures accurately and consistently	3.5	3	3	3	3.5	3
17. The indicator has sufficient discriminative power	3	2.5	3	3	3	3
18. The indicator has been piloted in practice	4	1	2	4	4	4
19. The efforts needed for data collection have been considered	3.5	2	2.5	3	3	3.5
20. Specific instructions for presenting and interpreting the indicator results are provided	3.5	3	3	3	3	4

AIRE, Appraisal of Indicators through Research and Evaluation.

## Discussion

The primary objective of this systematic review was to identify quality indicators specific to outpatient palliative care. Previous systematic reviews have primarily focused on general palliative care quality indicators or specific domains, such as quality indicators for palliative care in intensive care units.^23^^,^^35^ This study makes a significant contribution as the first systematic review dedicated solely to identifying quality indicators for outpatient palliative care.

In this review, six studies were identified, comprising 32 unique indicators specific to outpatient palliative care. These indicators primarily address the process and outcome aspects of care, with limited attention to structural aspects. Based on our categorization, the identified indicators fall into four main categories: Patient–Provider Relationship and Mutual Understanding, Symptom and Pain Management, Supportive Care and Referrals, and Overall Patient Experience Evaluation. Our findings highlight a notable scarcity of quality indicators tailored to outpatient palliative care, underscoring a gap in the literature and the pressing need for further research in this area.

Unlike inpatient care, where providers have continuous opportunities to build relationships, the outpatient setting demands efficient and meaningful communication.^36^ The five indicators in the Patient–Provider Relationship and Mutual Understanding category emphasize the importance of ensuring that patients are perceived and respected as unique individuals while addressing their emotional and psychosocial needs. For instance, indicators such as “feeling seen as a person, not just someone with a medical problem” highlight the significance of humanizing care, particularly in outpatient settings where patient autonomy and perspectives are central. These indicators also help foster trust, which is essential for the successful long-term management of palliative care needs.^37^ In outpatient care, where providers often depend on patients to report changes in their condition, establishing strong relationships is critical for proactively identifying and addressing issues.

The five indicators in the Supportive Care and Referrals category underscore the planning required to ensure patients receive appropriate care during critical stages of their illness. For example, indicators like “outpatient referral of advanced cancer patients to inpatient palliative care within six months of expected death” highlight the importance of early integration of palliative care to improve the quality of end-of-life care. In outpatient settings, where patients often transition between primary care, specialized care, and home care, seamless referrals and coordinated care planning are vital.^38^ Communication plans following chemotherapy—another key aspect of this category—ensure that high-risk symptoms, such as nausea and diarrhea, are effectively managed to prevent unnecessary suffering or complications. Collectively, these indicators stress the importance of a well-coordinated care network in outpatient settings, where fragmented care can lead to significant negative outcomes.

The Overall Patient Experience Assessment category captures the patient's perspective on the quality of outpatient palliative care. The eight indicators in this category focus on subjective measures of care quality, such as “feeling heard and understood by the care team” and “perceiving that the provider and care team prioritize individual interests.” These indicators are particularly important in outpatient settings, where the episodic nature of care may sometimes leave patients feeling unsupported or disconnected from their care team.^39^ By prioritizing the patient's voice, these metrics provide valuable insights into how well patients' needs are being met beyond clinical outcomes. Additionally, indicators like “receiving the expected help for pain in the past six months” offer critical feedback on whether patients feel their concerns are adequately addressed, fostering a sense of partnership and trust. In outpatient care, where patients and caregivers often bear the responsibility of managing day-to-day challenges,^40^ addressing these aspects of care quality is essential to enhancing overall satisfaction and emotional well-being.

The studies included in this review primarily emphasized physical symptom management, particularly pain, as well as the importance of patients feeling heard and understood. These areas are especially critical in outpatient settings for several reasons. Effective pain management is a cornerstone of palliative care and significantly impacts the quality of life for patients with life-limiting illnesses.^41^ In outpatient environments, where patients often manage their conditions at home with limited supervision from health care providers, the ability to control pain and other physical symptoms effectively is crucial.^42^ Ensuring that patients feel heard and understood is equally important, as it enhances satisfaction, strengthens trust between patients and providers, and supports better adherence to treatment plans.^43^ Addressing these areas helps establish a patient-centered approach to care, which is vital in outpatient settings, where continuity of care and the ability for patients to express their needs and concerns play a pivotal role in the overall effectiveness of palliative care services.^44^

The absence of quality indicators addressing cultural and social aspects in the reviewed studies highlights a critical gap in the current understanding and implementation of outpatient palliative care. Cultural awareness in palliative care ensures that the diverse values, beliefs, and practices of patients and their families are respected and integrated into care plans.^45^ This is especially important in outpatient settings, where patients often come from diverse cultural backgrounds and may have unique needs. Addressing cultural aspects can improve communication, build trust, and foster a more supportive care environment.^46^

Similarly, social factors such as support networks, access to community resources, and the social context of patients play a significant role in effective palliative care. These factors influence patients’ well-being, access to care, and ability to manage their illness.^47^ Incorporating cultural and social dimensions into outpatient palliative care can help providers better address the comprehensive needs of patients, ultimately improving their quality of life and ensuring that care is equitable, holistic, and patient-centered.

The generalizability of these findings to other settings, including Iran and specifically Tehran, warrants careful consideration. The religious, cultural, economic, and health care system contexts in Iran differ significantly from those in countries where the included studies were conducted. In Iran, religious beliefs deeply influence perceptions of illness, end-of-life care, and decision-making processes,^48^ which may affect how certain quality indicators are prioritized or interpreted in outpatient palliative care. Additionally, economic constraints and disparities in health care infrastructure, including limited access to specialized outpatient palliative services and a shortage of trained personnel,^49^ present challenges for implementing some of the identified indicators.

The health care system of Iran, which places a strong emphasis on family involvement in care,^50^ may also necessitate adapting indicators to reflect the central role of families in decision-making and caregiving. Furthermore, community-based and charitable organizations often play a crucial role in supporting outpatient palliative care services in Iran,^51^ which may shift priorities toward indicators that assess social and community support structures. Considering these factors, it is essential to locally adapt, validate, and prioritize these quality indicators to ensure they align with the unique values, expectations, and resource availability of the Iranian health care environment.

The lack of quality indicators specific to outpatient palliative care further underscores the need for dedicated research to identify indicators tailored to this setting. Outpatient palliative care differs significantly from other care environments, such as hospitals or intensive care units, in terms of patient needs, available resources, and care delivery models.^13^ Therefore, developing indicators that capture the unique complexities and challenges of outpatient palliative care is essential.

Focusing on the specific characteristics of outpatient settings is important for several reasons. First, understanding the distinct features and challenges of outpatient care can help researchers and health care professionals design targeted interventions and quality improvement initiatives. Second, acknowledging the diversity within outpatient palliative care—such as variations in patient populations, disease trajectories, and care delivery contexts—can facilitate the development of tailored quality indicators. Finally, a greater focus on outpatient settings fosters a comprehensive understanding of care quality in these environments, enabling meaningful comparisons across studies and health care institutions.

Despite the geographic concentration of available studies, this systematic review makes a distinct and valuable contribution to the field by being the first to comprehensively synthesize outpatient palliative care quality indicators and organize them within a structured framework. This structured categorization highlights not only the priority areas currently emphasized in outpatient palliative care but also exposes critical gaps — particularly in cultural, social, and structural domains — that have received limited attention. By systematically mapping the available indicators and identifying these gaps, our study provides a clear foundation for future research and guideline development aimed at improving the quality of outpatient palliative care. Furthermore, this review offers a starting point for health care providers and policymakers in other contexts to evaluate the applicability of these indicators within their own health care systems and to initiate local adaptation and validation efforts.

### Implications for nursing practice and research

The findings of this review offer important guidance for nursing practice in outpatient palliative care settings. By highlighting key quality indicators, nurses can better assess and improve care processes, strengthen communication with patients and families, and address physical and emotional needs more effectively. Incorporating these indicators into routine practice can enhance patient-centered care, ensure symptom relief, and improve overall patient satisfaction. From a research perspective, these findings highlight gaps in current indicators, particularly in the areas of structural, cultural, and social dimensions of care. Future research should focus on developing and validating additional indicators that reflect these dimensions, as well as exploring the implementation and impact of quality indicators in diverse clinical settings.

### Limitations

This systematic review has several limitations that should be considered when interpreting its findings. First, the search was limited to articles published in English, which may have excluded relevant studies published in other languages, potentially introducing language bias. Second, the relatively small number of studies included in this review may restrict the generalizability of the findings to diverse outpatient palliative care settings. Additionally, the majority of identified indicators were process-based, which may limit the comprehensiveness of the quality assessment by underrepresenting structural and outcome aspects of care. Furthermore, reliance on published studies introduces a risk of publication bias, as studies with positive results are more likely to be published.

Another important limitation of this review is the geographic concentration of the included studies, all of which originated from the United States. This geographic bias may significantly affect the generalizability of the identified quality indicators to health systems with different structures, resources, cultural norms, and policy priorities. The U.S. health care system has unique characteristics, including a high degree of specialization, market-driven care models, and specific regulatory frameworks,^52^ which may influence the types of indicators considered important and feasible. In settings with publicly funded or mixed health care systems, or where community-based palliative care plays a larger role, different quality priorities may emerge. Moreover, the cultural values embedded within U.S.-developed indicators may not fully capture priorities in other societies, particularly in non-Western or resource-limited settings. This highlights the need for further international research to develop, adapt, and validate outpatient palliative care quality indicators that reflect local health care delivery models, cultural expectations, and resource constraints.

## Conclusions

This systematic review identified a limited number of quality indicators specific to outpatient palliative care, revealing significant gaps in the existing literature. While some indicators address physical and process-related aspects of care, there is a notable absence of indicators that account for structural, cultural, and social dimensions. This highlights the critical need for further research to develop comprehensive quality indicators that encompass all facets of palliative care. Addressing these gaps is essential for improving the quality, equity, and patient-centeredness of outpatient palliative care services. Future studies should prioritize the development of structural, cultural, and social indicators to ensure care delivery is holistic and inclusive. By addressing the diverse needs of patients, health care providers can enhance patients’ quality of life and advance the effectiveness and equity of outpatient palliative care.

## CRediT authorship contribution statement

Sina Sharifi: Conceptualization, Data curation, Investigation, Methodology, Writing – original draft, and Writing – review & editing. Qasem Mousavi: Conceptualization, Investigation, Writing – original draft, and Writing – review & editing. Salman Barasteh: Investigation; Methodology, Conceptualization, Writing – original draft, and Writing – review & editing. Akram Parandeh: Methodology, Writing – original draft, and Writing – review & editing. All authors have read and approved the final manuscript.

## Ethics statement

The study was approved by the Institutional Ethics Committee of Baqiyatallah University of Medical Sciences (Approval No. IR.BMSU.BAQ.REC.1403.067).

## Data availability statement

Data availability is not applicable to this article as no new data were created or analyzed in this study.

## Declaration of generative AI and AI-assisted technologies in the writing process

No AI tools/services were used during the preparation of this work.

## Funding

This study received no external funding.

## Declaration of competing interest

The authors declare no conflict of interest.

## References

[1] Radbruch L., De Lima L., Knaul F. (2020). Redefining palliative care—a new consensus-based definition. J Pain Symptom Manag.

[2] Watson M., Campbell R., Vallath N., Ward S., Wells J. (2019).

[3] Etkind S.N., Bone A.E., Gomes B. (2017). How many people will need palliative care in 2040? Past trends, future projections and implications for services. BMC Med.

[4] Silva TCd, Nietsche E.A., Cogo S.B. (2021). Palliative care in primary health care: an integrative literature review. Rev Bras Enferm.

[5] Hui D., Bruera E. (2020). Models of palliative care delivery for patients with cancer. J Clin Oncol.

[6] Schoenherr L.A., Bischoff K.E., Marks A.K., O'Riordan D.L., Pantilat S.Z. (2019). Trends in hospital-based specialty palliative care in the United States from 2013 to 2017. JAMA Netw Open.

[7] Reymond L., Parker G., Gilles L., Cooper K. (2018). Home-based palliative care. Aust J Gen Pract.

[8] Shah R., Georgousopoulou E.N., Al-Rubaie Z. (2022). Impact of ambulatory palliative care on symptoms and service outcomes in cancer patients: a retrospective cohort study. BMC Palliat Care.

[9] Rabow M., Kvale E., Barbour L. (Dec 2013). Moving upstream: a review of the evidence of the impact of outpatient palliative care. J Palliat Med.

[10] Zimmermann C., Buss M.K., Rabow M.W., Hannon B., Hui D. (2023). Should outpatient palliative care clinics in cancer centers be stand alone or embedded?. J Pain Symptom Manag.

[11] Cunningham C., Ollendorf D., Travers K. (2017). The effectiveness and value of palliative care in the outpatient setting. JAMA Intern Med.

[12] Finlay E., Rabow M.W., Buss M.K. (2018). Filling the gap: creating an outpatient palliative care program in your institution. Am Soc Clin Oncol Educ Bk.

[13] Kathryn Hallman M. (2019). Outpatient palliative care. Clin J Oncol Nurs.

[14] Meier D.E., Beresford L. (2008). Outpatient clinics are a new frontier for palliative care. J Palliat Med.

[15] Teno J.M., Price R.A., Makaroun L.K. (2017). Challenges of measuring quality of community-based programs for seriously ill individuals and their families. Health Aff.

[16] Oliver D.J. (2018). Improving patient outcomes through palliative care integration in other specialised health services: what we have learned so far and how can we improve?. Ann Palliat Med.

[17] Wentlandt K., Seccareccia D., Kevork N. (2016). Quality of care and satisfaction with care on palliative care units. J Pain Symptom Manag.

[18] Oosterveld-Vlug M., Custers B., Hofstede J. (2019). What are essential elements of high-quality palliative care at home? An interview study among patients and relatives faced with advanced cancer. BMC Palliat Care.

[19] Bickel K.E., McNiff K., Buss M.K. (2016). Defining high-quality palliative care in oncology practice: an American society of clinical oncology/American academy of hospice and palliative medicine guidance statement. J Oncol Pract.

[20] Báo A.C.P., Amestoy S.C. (2019). Moura GMSSd, Trindade LdL. Quality indicators: tools for the management of best practices in Health. Rev Bras Enferm.

[21] Ramalho A., Castro P., Goncalves-Pinho M. (2019). Primary health care quality indicators: an umbrella review. PLoS One.

[22] Rabow M.W., O'Riordan D.L., Pantilat S.Z. (2014). A statewide survey of adult and pediatric outpatient palliative care services. J Palliat Med.

[23] Tanaka Y., Masukawa K., Kawashima A., Hirayama H., Miyashita M. (2023). Quality indicators for palliative care in intensive care units: a systematic review. Ann Palliat Med.

[24] Dy S.M., Kiley K.B., Ast K. (2015). Measuring what matters: top-ranked quality indicators for hospice and palliative care from the American Academy of hospice and palliative medicine and hospice and palliative nurses association. Article. J Pain Symptom Manag.

[25] Page M.J., McKenzie J.E., Bossuyt P.M. (2021). The PRISMA 2020 statement: an updated guideline for reporting systematic reviews. Int J Surg.

[26] de Koning J., Smulders A., Klazinga N. (2006). The Appraisal of Indicators through Research and Evaluation (AIRE) instrument. Amsterdam: Acad Med Cent.

[27] Assarroudi A., Heshmati Nabavi F., Armat M.R., Ebadi A., Vaismoradi M. (Feb 2018). Directed qualitative content analysis: the description and elaboration of its underpinning methods and data analysis process. J Res Nurs.

[28] Donabedian A. (2005). Evaluating the quality of medical care. Milbank Q.

[29] Campion F.X., Larson L.R., Kadlubek P.J., Earle C.C., Neuss M.N. (2011). Advancing performance measurement in oncology: quality oncology practice initiative participation and quality outcomes. J Oncol Pract.

[30] Dy S.M., Lorenz K.A., Oneill S.M. (2010). Cancer quality-ASSIST supportive oncology quality indicator set feasibility, reliability, and validity testing. Cancer.

[31] Lorenz K.A., Dy S.M., Naeim A. (2009). Quality measures for supportive cancer care: the cancer quality-ASSIST project. Article. J Pain Symptom Manag.

[32] Rollison J., Bandini J.I., Gilbert M., Phillips J., Ahluwalia S.C. (Feb 2022). Incorporating the patient and caregiver voice in palliative care quality measure development. J Pain Symptom Manag.

[33] Walling A.M., Ast K., Harrison J.M. (2023). Patient-reported quality measures for palliative care: the time is now. J Pain Symptom Manag.

[34] Ahluwalia S.C., Vegetabile B.G., Edelen M.O. (Jun 2022). MACRA palliative care quality measure development-testing summary report: measure name: feeling heard and understood. Rand Health Q.

[35] De Roo M.L., Leemans K., Claessen S.J. (2013). Quality indicators for palliative care: update of a systematic review. J Pain Symptom Manag.

[36] Prip A., Møller K.A., Nielsen D.L., Jarden M., Olsen M.-H., Danielsen A.K. (2018). The patient–healthcare professional relationship and communication in the oncology outpatient setting: a systematic review. Cancer Nurs.

[37] Murahashi M., Tamba K., Takanashi T. (2024). Bereaved family caregivers perception of trust in palliative care doctors by patients with terminal cancer. J Soc Work End-of-Life Palliat Care.

[38] Kansagara D., Chan B., Harmon D., Englander H. (2013). Transitions of care: putting the pieces together. AMA J Ethics.

[39] Wolper L.F. (2010). Health care administration: managing organized delivery systems: managing organized delivery systems. Jones & Bartlett Learn.

[40] Sallie G., Golia De, Wang R. (2023).

[41] Krakauer E.L., Kwete X., Verguet S. (2018).

[42] Person L., Taylor E.J. (2002). Managing Pain in Outpatients: there are particular challenges to pain control in outpatient settings. AJN Am J Nurs.

[43] Ingersoll L.T., Saeed F., Ladwig S. (2018). Feeling heard and understood in the hospital environment: benchmarking communication quality among patients with advanced cancer before and after palliative care consultation. J Pain Symptom Manag.

[44] Öhlén J., Carlsson G., Jepsen A., Lindberg I., Friberg F. (2016). Enabling sense-making for patients receiving outpatient palliative treatment: a participatory action research driven model for person-centered communication. Palliat Support Care.

[45] Mathew-Geevarughese S.E., Corzo O., Figuracion E. (2019). Cultural, religious, and spiritual issues in palliative care. Prim Care Clin Off Pract.

[46] Cain C.L., Surbone A., Elk R., Kagawa-Singer M. (2018). Culture and palliative care: preferences, communication, meaning, and mutual decision making. J Pain Symptom Manag.

[47] Bradley N., Lloyd-Williams M., Dowrick C. (2018). Effectiveness of palliative care interventions offering social support to people with life-limiting illness—a systematic review. Eur J Cancer Care.

[48] Fallahi S., Rassouli M., Mojen L.K. (2017).

[49] Eshghi P, Noripour S, Momtazmanesh N, Mojen LK. Development of palliative care for children with cancer in Iran: a local model. Int J Cancer Manag. 18(1).

[50] Jafarpoor H., Parvaneh V., Manoochehri H. (2020). How is family involved in clinical care and decision-making in intensive care units? A qualitative study. Contemp Nurse.

[51] Ansari M.P.C., Rassouli M.P., Akbari M.M., Abbaszadeh A.P., Akbari Sari A.P. (Apr 2018). Educational needs on palliative care for cancer patients in Iran: a SWOT analysis. Int J Community Based Nurs Midwifery.

[52] Birn A.E., Hellander I. (Mar 2016). Market-driven health care mess: the United States. Cad Saude Publica.

