# Supplementary material for: Quality indicators of outpatient palliative care: A systematic review
Source: Asia Pac J Oncol Nurs. 2025 May 10;12:100718. doi: 10.1016/j.apjon.2025.100718 (PMC12151211; doi:10.1016/j.apjon.2025.100718)
Supplement: Multimedia component 1 [file mmc1.docx]

Table S1. Database search terms used in the systematic review.

| Aspect | Search terms |
| --- | --- |
| Quality Measures | "Quality Indicator" OR "Quality Measure" OR "Quality Criterium" OR "Quality Assessment" OR "Care Performance" OR "Outcome Measure", "Quality Criteria" |
| Type of Care | "Palliative Care" OR " Palliative" OR "Terminal Care" OR "Life Support Care" OR "Hospice" OR "End of Life Care" |
| Care Setting | "Outpatient Care" OR "Outpatient Services" OR "Outpatient Service" OR "Ambulatory" OR "Outpatient" |
